# Neural Biomarkers Distinguish Severe From Mild Autism Spectrum Disorder Among High-Functioning Individuals

**DOI:** 10.3389/fnhum.2021.657857

**Published:** 2021-05-06

**Authors:** Di Chen, Tianye Jia, Yuning Zhang, Miao Cao, Eva Loth, Chun-Yi Zac Lo, Wei Cheng, Zhaowen Liu, Weikang Gong, Barbara Jacquelyn Sahakian, Jianfeng Feng

**Affiliations:** ^1^Institute of Science and Technology for Brain-Inspired Intelligence, Fudan University, Shanghai, China; ^2^Key Laboratory of Computational Neuroscience and Brain-Inspired Intelligence, Fudan University, Ministry of Education, Shanghai, China; ^3^Centre for Population Neuroscience and Precision Medicine, MRC SGDP Centre, IoPPN, King’s College London, London, United Kingdom; ^4^State Key Laboratory of Cognitive Neuroscience and Learning, Beijing Normal University, Beijing, China; ^5^School of Psychology, University of Southampton, Southampton, United Kingdom; ^6^Sackler Institute for Translational Neurodevelopment, Department of Forensic and Neurodevelopmental Sciences, IoPPN, King’s College London, London, United Kingdom; ^7^Department of Psychiatry, School of Clinical Medicine, University of Cambridge, Cambridge, United Kingdom; ^8^School of Mathematical Sciences and Centre for Computational Systems Biology, Fudan University, Shanghai, China; ^9^Department of Computer Science, University of Warwick, Coventry, United Kingdom

**Keywords:** autism spectrum disorder, functional magnetic resonance imaging, high-functioning autism, neural biomarker, autism diagnostic observation schedule

## Abstract

Several previous studies have reported atypicality in resting-state functional connectivity (FC) in autism spectrum disorder (ASD), yet the relatively small effect sizes prevent us from using these characteristics for diagnostic purposes. Here, canonical correlation analysis (CCA) and hierarchical clustering were used to partition the high-functioning ASD group (i.e., the ASD discovery group) into subgroups. A support vector machine (SVM) model was trained through the 10-fold strategy to predict Autism Diagnostic Observation Schedule (ADOS) scores within the ASD discovery group (*r* = 0.30, *P* < 0.001, *n* = 260), which was further validated in an independent sample (i.e., the ASD validation group) (*r* = 0.35, *P* = 0.031, *n* = 29). The neuroimage-based partition derived two subgroups representing severe versus mild autistic patients. We identified FCs that show graded changes in strength from ASD-severe, through ASD-mild, to controls, while the same pattern cannot be observed in partitions based on ADOS score. We also identified FCs that are specific for ASD-mild, similar to a partition based on ADOS score. The current study provided multiple pieces of evidence with replication to show that resting-state functional magnetic resonance imaging (rsfMRI) FCs could serve as neural biomarkers in partitioning high-functioning autistic individuals based on their symptom severity and showing advantages over traditional partition based on ADOS score. Our results also indicate a compensatory role for a frontocortical network in patients with mild ASD, indicating potential targets for future clinical treatments.

## Introduction

Autism spectrum disorder (ASD) is a neurodevelopmental condition characterized by qualitative impairment in social communication, as well as restricted and repetitive behaviors (American Psychiatric Association, 2013), and affects approximately 1% of children globally (Kim et al., 2011; Baio et al., 2018). Although more recent conceptualization of ASD considers symptoms on a spectrum ranging from mild to severe, a categorical stratification of ASD based on the severity of symptom presentation is often made for diagnostic purposes (Lord et al., 2000). Currently, ASD diagnosis is entirely guided by behavioral indices, which has been criticized for its high heterogeneity in both phenotypic presentation and etiology (Ecker and Murphy, 2014). For example, a large portion of children with ASD (11–60%) also have mild intellectual disability, i.e., intelligence quotient (IQ) lower than 70 (Baio et al., 2018; Lord et al., 2018), largely attributing to a range of rare *de novo* mutations (Sanders et al., 2015; Weiner et al., 2017). The recent development in neuroscience has provided insights into the potential of neural biomarkers to characterize ASD (Ecker and Murphy, 2014; Eilam-Stock et al., 2014; Cheng et al., 2015, 2017; Jack and Pelphrey, 2017; Holiga et al., 2019), e.g., identifying the middle temporal, prefrontal, and parietal areas (Cheng et al., 2015; Holiga et al., 2019) as hub regions enriched with functional connectivities (FCs) that distinguish autistic patients from controls. However, no previous MRI study has found effect sizes large enough to indicate that brain structure or function could be used as a diagnostic marker. This has prompted a shift to focus on the identification of stratification biomarkers to parse this heterogeneous condition into more homogeneous subgroups (Loth et al., 2016). Previous studies have explored the use of neural features from fMRI data to identify subgroups of autistic patients (Groen et al., 2010; Lombardo et al., 2018), although few of them have directly targeted the diagnostic scales, such as Autism Diagnostic Observation Schedule (ADOS). Moreover, few studies have evaluated the agreement between biomarker-based stratification of ASD patients and differences in clinical symptom profile or severity.

In this study, we first investigated if resting-state functional brain networks could be used as stratification and prediction biomarkers for the severity of autism (measured by ADOS) among a group of high-functioning autistic participants (IQ ≥ 70) (Baio et al., 2018; Lord et al., 2018) using a series of multivariate statistical approaches including canonical correlation analysis (CCA), hierarchical clustering (Jia et al., 2016; Drysdale et al., 2017), and support vector machine (SVM) (Chang and Lin, 2011). We then further investigated specific neural biomarkers among stratified ASD groups, as well as subgroups of ASD compared with control.

## Materials and Methods

### Functional Connectivity Data Preprocessing

The study samples were derived from the Autism Brain Imaging Data Exchange (ABIDE) I and II (Di Martino et al., 2014), released in 2012 and 2016, respectively. Approval was required by the respective site Institutional Review Board (IRB). All participants’ autistic symptoms (measured using ADOS), as well as resting-state functional magnetic resonance imaging (rsfMRI) data, were collected from multiple data acquisition sites across the globe. More details on data acquisition can be found on the initiative’s website^1^.

The rsfMRI data were preprocessed following a standard pipeline^2^, which included slice timing correction, motion correction, spatial smoothing (full-width half maximum = 6 mm), despiking motion artifacts using the BrainWavelet Toolbox (Patel et al., 2014; Patel and Bullmore, 2016), registering to MNI152 standard space with a voxel size of 2 mm × 2 mm × 2 mm by first aligning the functional image to the individual T1 structural image using boundary-based registration (Greve and Fischl, 2009) and then to standard space by FSL’s tool FLIRT^3^ and FNIRT^4^; nuisance covariates including Friston 24 head motion parameters (Friston et al., 1996), white matter signal, cerebrospinal fluid signal (obtained by FSL’s tool FAST^5^), and global signal were regressed out from the blood oxygen level-dependent (BOLD) signal and band-pass filtering (0.01–0.1 Hz) by AFNI (Cox, 1996). The processed imaging data of all subjects were visually checked for quality control.

The 2nd edition of the Automated Anatomical Labeling Atlas (AAL-2) template (Rolls et al., 2015) was used to parcellate the brain into 94 regions of interest (ROIs) (Supplementary Table 1) (Cheng et al., 2019). The time series were then extracted in each ROI by averaging the signals of all voxels included, leading to 94 functional nodes spanning across the brain. For each pair of nodes in this brain pairwise analysis, their Pearson correlation coefficients were calculated followed by the Fisher *z*-transformation of FCs. Finally, standardized *z*-scores were calculated for each subject’s FCs to make them comparable across subjects and sites (Supplementary Figures 1, 2). Therefore, for each subject, the constructed brain network consisted of 94 brain regions and 4,371 (i.e., $\frac{94\times 93}{2}=4,371$) FCs.

### Sample Inclusive/Exclusive Criteria

Subjects selected for the current investigation included those who (i) had a full IQ score equal or exceeding 70, (ii) mean framewise displacement did not exceed 0.5 mm, and (iii) aged 6–30 years at the time of assessment. To match our statistical analysis purposes, we selected participants and divided them into three groups: the ASD group, the control group, and the independent ASD validation group. The ASD group was selected using the following inclusion criteria: (i) had a diagnosis of autism in either ABIDE I or II and (ii) had an ADOS total score higher than seven following the diagnostic recommendation (Lord et al., 2000), including Asperger’s and pervasive developmental disorder not otherwise specified [i.e., in line with the term ASD as described in the *Diagnostic and Statistical Manual of Mental Disorders* (Fifth Edition) (DSM-V)]. Note that ADOS total score was calculated differently in one study site than the rest. Therefore, we re-calculated ADOS total score across all sites to be the additive score of the ADOS Communication subscale score and ADOS Social Interaction subscale score following suggestions Lord et al. (2000) (Supplementary Table 2); and (iii) from sites with three or more ASD subjects who met the same criteria, to avoid unreliable estimation of site effect.

The ASD patients were further separated into a discovery group, which has complete information for all ADOS subscales mandated for the fMRI feature selection in the training procedure, and a validation group, which has at least one subscale of ADOS missing, thus only serving as the testing sample. The control group of participants was selected from the sites where the ASD group was also selected. Each control subject was recorded as “healthy control” in either ABIDE I or II. The independent ASD validation group consisted of participants who were (i) diagnosed with ASD without a pre-calculated ADOS total score and (ii) from sites that have not been selected in either of the two testing samples.

As a result of subject selection and quality control, 260 participants in the ASD discovery group, 574 in the control group, and 29 in the ASD validation group were included in the current study. Demographic information is summarized in Table 1. There is no difference in IQ, gender ratio, and age between the ASD discovery and ASD validation groups. Confounding factors such as the full IQ, gender, age, mean framewise displacement, and site were regressed out in the following analysis unless otherwise specified. The methods were implemented using the software MATLAB [Version: 9.5.0.944444 (R2018b)].

**TABLE 1:** Sample characteristics.

	ASD-discovery	Healthy controls	ASD-validation	ASD-discovery vs. control	ASD-discovery vs. ASD-validation
Sample size	260	574	29		
Full IQ	105.15 (16.28)	114.43 (12.63)	108.48 (16.87)	*t* = −8.95;	*t* = −1.04;
Mean (SD)				Cohen’s *D* = −0.67;	Cohen’s *D* = −0.20;
				*P* < 0.001	*P* = 0.298
Age:	15.24 (5.83)	13.16 (5.10)	16.91 (3.01)	*t* = 5.22;	*t* = −1.52;
Mean (SD)				Cohen’s *D* = 0.39;	Cohen’s *D* = −0.30;
				*P* < 0.001	*P* = 0.130
Male %	91.15%	70.56%	93.10%	*P* < 0.001	*P* = 0.724

*ASD, autism spectrum disorder.*

### Statistical Analysis

**FIGURE 1:**
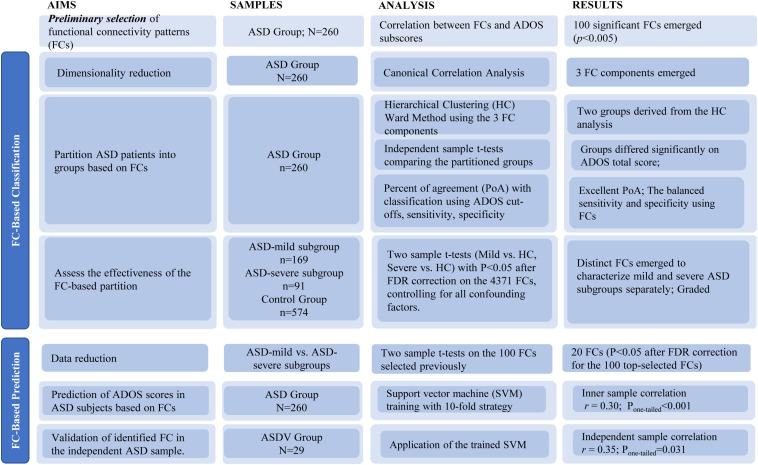
Flowchart of study aims, methods, and results.

Detailed data analysis strategy is outlined in Figure 1. Prior to statistical modeling, we calculated Spearman’s rank correlation matrices between 4,371 FCs and three ADOS subscales (communication, social interaction, and restricted/stereotyped behaviors) among participants from the ASD discovery group. Only FCs in Spearman’s rank correlation (threshold *P* < 0.005) with at least one ADOS subscales were included, which was used to reduce the number of FCs entered into the CCA.

The above threshold *P* < 0.005 was set to select a sufficient amount of but not too many FCs. While there is no gold standard to choose such a threshold, it has been suggested that too stringent a threshold is likely to omit informative features, while an over-relaxed one will end up with too many non-informative FCs as well as risking for serious overfitting problems. The detailed calculations of selected FCs based on different thresholds of *P*-values are summarized in Supplementary Table 3.

We investigated the effectiveness of using functional networks to differentiate mild from severe high-functioning ASD patients in two ways.

(1) FC-based stratification of high-functioning ASD patients and its effectiveness to differentiate symptom severity.

First, CCA was applied to further reduce the remaining FCs derived above into three orthogonal components that maximized the explained variance of three ADOS subscales. Due to the inevitable overfitting of the combination of the FC selection process and CCA, the *P*-values of the overall correlation (measured by η^2^, i.e., 1 − Wilk’s λ) and each component correlation were assessed through a permutation test (Dinga et al., 2019); at each of the 10,000 iterations, the ADOS subscales were randomly shuffled, and the whole process (including selecting the same number of top FCs to keep feature to sample radio based on its Spearman’s rank correlation with ADOS subscales to a matched number) was re-conducted, thus providing an empirical null distribution of each CCA statistic, i.e., η^2^ and component correlations, which accounted for the overfitting due to both the FC selection process and the CCA. Based on the same permutation process described above, we also provided an adjusted η^2^ (i.e., adj-η^2^) that accounted for the inflation of η^2^ due to the overfitting of both the FC selection process and the CCA (Jia et al., 2020).

Next, the significant FC components were entered into a hierarchical clustering analysis with Euclidean distance and the Ward method. Note that due to the nature of the hierarchical clustering algorithm, the two-cluster partition serves as a default option that further clustering makes no sense unless the two-cluster partition is meaningful, which could be confirmed if the ADOS total scores of the two partitions from the hierarchical clustering were significantly different using a two-sample *t*-test. Note that the corresponding *P*-values were again assessed by using the permutation process described above (i.e., re-conducting all processes, including the FC selection, CCA, and hierarchical clustering at each of 10,000 iterations) to establish the null distribution of the two-sample *t*-statistic where no real partition was present. In addition, we also investigated if further clustering (i.e., from 3 to 10) could be superior over the default choice of two through the silhouette method (Rousseeuw, 1987). To further evaluate if the partitioned subgroups were of clinical relevance, we assessed the agreement between our FC-based nonparametric stratification and the traditional behavior-based parametric classification of severe versus mild ASD patients based on chosen cutoffs of the ADOS total score, i.e., the percentage of agreement (PoA), the true positive rate to identify a severe ASD patient (sensitivity) and the true negative rate to identify a mild ASD patient (specificity). Please note that due to the lack of known gold standard for the severe versus mild partition of ASD patients, we calculated two sets of receiver operating characteristic (ROC) curves, i.e., one with the FC-based nonparametric partition as the reference and one with the partition at each ADOS cutoff as the reference.

The last step in this set of analyses involved comparing the two ASD discovery subgroups (i.e., severe and mild in ASD) against the control group on the 4,371 FCs using two-sample *t*-tests [*P* < 0.05, false discovery rate (FDR) correction], to investigate if the patterns of differentiated FCs among the three groups (with the hypothesis of a graded change of FC strengths from the control, through mild, to severe groups; i.e., it is the same FCs with similar effects that could distinguish the severe ASD group from the mild ASD group, and the mild ASD group from the control group). Specifically, we first multiplied −1 to those FCs with a negative *t*-statistics; i.e., FCs were found smaller in the severe or mild ASD groups than in the control group. Such a process simplified the testing hypothesis into a graded increase of FCs from the control, through mild, to severe groups; Then, we summed up all hence derived FCs in each group (i.e., the control, mild, or severe group); and the *t*-tests between paired groups, i.e., “severe versus mild” and “mild versus control,” were calculated to investigate if the given hypothesis of graded change was satisfied. The graded change in each individual FC was also investigated.

The same procedure with the FCs identified in the above analysis was also conducted between partitions derived based on given thresholds of ADOS total scores.

(2) FC-based prediction of ADOS scores in high-functioning ASD patients.

First, we further selected the statistically significant FCs (*P* < 0.05 after FDR) among the initially selected top FCs by assessing the significance of differences between the ASD discovery mild and severe subgroups derived above. We then trained an SVM model [LIBSVM: library for SVMs (Cortes and Vapnik, 1995; Chang and Lin, 2011); SVM type: multi-class classification; kernel type: radial basis function] in the ASD discovery group (*n* = 260) to predict each individual’s ADOS total scores, using a 10-fold strategy. Iteratively, the SVM model was trained with 234 out of 260 subjects to predict the leftover 26 subjects’ ADOS total score. After 10 iterations, the Pearson correlation between the predicted and observed ADOS total scores (*n* = 260) was then calculated to evaluate the accuracy of prediction. To alleviate the issue of overfitting, we omitted the step of parameter selection by using the default setting of the SVM model instead (Chang and Lin, 2011). The SVM model trained above was then validated in the ASD validation group (*n* = 29, leftover individuals due to incomplete subscale information). The flowchart of validation could be found in Supplementary Figure 3.

## Results

### Functional Connectivity-Based Stratification of High-Functioning ASD Patients and Its Effectiveness to Differentiate Symptom Severity

**FIGURE 2:**
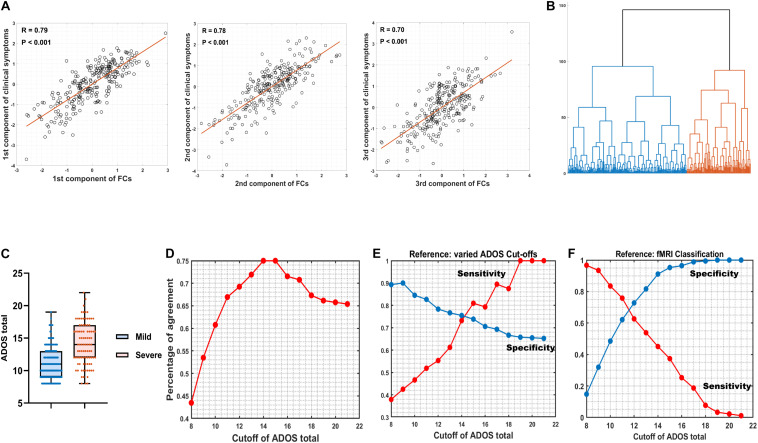
Canonical correlation analysis (CCA) and hierarchical clustering. **(A)** The relation between three combinations of functional connectivities (FCs) and three clinical symptom combinations, respectively (*r* > 0.7, *P*_permutation_ < 0.001). **(B)** A hierarchical clustering (method: ward) partitioned the high-functioning autism spectrum disorder (ASD) group into two subgroups (*n*_*mild*_ = 169, *i*_*severe*_ = 91). **(C)** Box figure of Autism Diagnostic Observation Schedule (ADOS) total score in the two ASD subgroups. The ASD-severe subgroup (*n* = 91) has significantly higher ADOS total scores (*t* = 9.23, *P*_permutation_ = 0.009) than the ASD-mild subgroup (*n* = 169). Full IQ, gender, age, and site were controlled for when calculating *t* and *P*-values. **(D)** Percentage of agreement between resting-state functional magnetic resonance imaging (rsfMRI) FC-based stratification and segregation based on varied cutoffs at ADOS total score. **(E)** True positive rate (sensitivity) and true negative rate (specificity) by setting varied ADOS cutoffs as the reference. **(F)** True positive rate (sensitivity) and true negative rate (specificity) by setting fMRI classification as the reference.

Spearman’s rank correlation identified 100 FCs in association with at least one out of three ADOS subscales (communication, social interaction, and restricted/stereotyped behaviors) with balanced (the analysis with different statistical thresholds; see Supplementary Table 3) *P* < 0.005 uncorrected. CCA revealed three orthogonal components that explained η^2^ = 92.66% (Wilk’s lambda = 0.0734, *P*_permutation_ < 0.001; adj-η^2^ = 60.93%) of the variance of across three ADOS sub-scores (*R*_1_ = 0.79, *P*_permutation_ < 0.001; *R*_2_ = 0.78, *P*_permutation_ < 0.001; *R*_3_ = 0.70, *P*_permutation_ < 0.001; the permutation process includes both the steps of re-selecting the top 100 FCs (i.e., maintaining feature to sample radio = 100/260 = 38.46%) and CCA at each iteration and therefore properly adjusted for any possible overfittings; see section “Materials and Methods”; Figure 2A).

A hierarchical clustering (method: Ward) based on the three components then partitioned the ASD discovery individuals (i.e., with complete information of ADOS subscales; see section “Materials and Methods”) into two subgroups (i.e., as the default choice of hierarchical clustering; Figure 2B) that were further found with significant differences on their ADOS total scores (*ASD-severe subgroup*: mean = 14.07, SD = 3.27, *n* = 91; *ASD-mild subgroup*: mean = 11.07, SD = 2.45, *n* = 169; Cohen’s *D* = 1.26, *t* = 9.23, *P*_permutation_ = 0.009; the permutation process includes the FC selection process, CCA, and the hierarchical clustering and therefore properly adjusted for any possible overfittings; see section “Materials and Methods”; Figure 2C), which thus confirmed the existence of two cluster. Follow-up analyses with silhouette scores, which calculated with different cluster numbers from 2 to 10, confirmed that the two-cluster partition was indeed the optimal choice (i.e., the maximal silhouette score for two-cluster, see Supplementary Table 4).

Our FC-based nonparametric partition of severe versus mild ASD discovery subgroups showed high agreements (PoA > 70% at cutoffs 13, 14, …, 17; Figure 2D and Supplementary Table 5) with segregations based on behavioral indices (i.e., the ADOS total score) using different cutoff scores. By setting varied ADOS cutoffs as the reference for the partition, the FC-based nonparametric partition showed highly consistent specificity (i.e., true negative rate > 0.65 for all cutoffs) and sensitivity (i.e., true positive rate > 0.70 at cutoffs larger than 14) (Figure 2E and Supplementary Table 5) in separating severe ASD patients from mild ones, indicating a highly stable performance of FC-based nonparametric partition even if the real classification is ambiguous. In contrast, by setting the FC-based nonparametric partition as the reference, a clear trade-off between specificity and sensitivity of identifying severe ASD patients was observed, with both larger than 0.60 only at ADOS total score of 11 and 12, i.e., with balanced sensitivity and specificity (Figure 2F and Supplementary Table 5), which is in line with the recommended clinical cutoff score (Lord et al., 2000).

**FIGURE 3:**
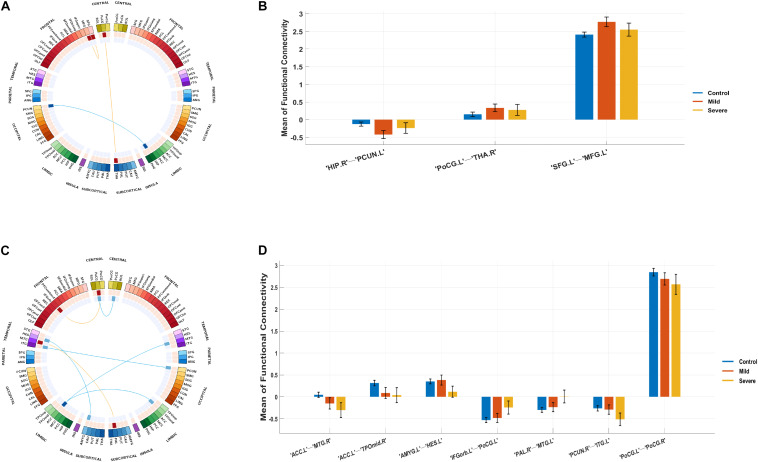
Biomarker (subgroups vs. controls) and its tendency. **(A)** Stratification candidate biomarker between the autism spectrum disorder (ASD)-mild subgroup and controls, corresponding to a threshold: false discovery rate (FDR) *P* < 0.05. **(B)** Three-group (controls, mild group, severe group) comparison on the ASD-mild specific functional connectivities (FCs). **(C)** Stratification candidate biomarker between the ASD-severe subgroup and controls, corresponding to a threshold (FDR *P* < 0.05). **(D)** Three-group (controls, mild group, severe group) comparison on the ASD-severe specific FCs.

Compared with the control group, the severe and mild ASD discovery subgroups indeed revealed two sets of distinct FCs characterizing each group separately (ASD-mild specific FCs: Figure 3A and Table 2A; ASD-severe specific FCs: Figure 3C and Table 2B). The ASD-mild specific FCs were stronger in the mild subgroup than those in the severe subgroup (*t* = 2.35, Cohen’s *D* = 0.32, *P* = 0.020), which were in turn stronger than the same FCs in the control group (*t* = 7.25, Cohen’s *D* = 0.65, *P* < 0.001) (Supplementary Table 6). In particular, FC between the left superior frontal gyrus (left SFG) and left middle frontal gyrus (left MFG) showed significant differences in both comparisons (severe vs. mild: *t* = −1.90, Cohen’s *D* = −0.26, *P*_one–tailed_ = 0.029; severe vs. control: *t* = 1.68, Cohen’s *D* = 0.19, *P*_one–tailed_ = 0.046; Figure 3B; Supplementary Table 7). The ASD-severe specific FCs revealed a graded difference among the three groups with the ASD-severe subgroup being the strongest and the control group the weakest (severe vs. mild: *t* = 4.63, Cohen’s *D* = 0.63, *P*_*two–tailed*_ < 0.001; mild vs. controls: *t* = 4.50, Cohen’s *D* = 0.40, *P*_two–tailed_ < 0.001; Supplementary Table 6), particularly in univariate FCs between the left anterior cingulate cortex (left ACC) and right middle temporal gyrus (right MTG) (severe vs. mild: *t* = −1.73, Cohen’s *D* = −0.24, *P*_one–tailed_ = 0.042; mild vs. controls: *t* = −2.25, Cohen’s *D* = −0.20, *P*_one–tailed_ = 0.012), and between the left orbital inferior frontal gyrus (left orbital IFG) and left postcentral gyrus (left PCG) (severe vs. mild: *t* = 2.43, Cohen’s *D* = 0.33, *P*_one–tailed_ = 0.008; mild vs. controls: *t* = 1.78, Cohen’s *D* = 0.16, *P*_one–tailed_ = 0.038) (Figure 3D; Supplementary Table 8).

**TABLE 2A:** Biomarkers between mild groups and controls.

Functional connectivity		*t*	Cohen’s *D*	*P*_two–tailed_
Region 1	Region 2			
Postcentral_L	Thalamus_R	4.38	0.39	1.39E−05
Frontal_Sup_2_L	Frontal_Mid_2_L	4.36	0.39	1.48E−05
Hippocampus_R	Precuneus_L	−4.25	−0.38	2.45E−05

**TABLE 2B:** Biomarker between severe groups and controls.

Functional connectivity		*t*	Cohen’s *D*	*P*_two–tailed_
Region 1	Region 2			
Amygdala_L	Heschl_L	–4.54	–0.52	6.79E–06
Cingulate_Ant_L	Temporal_Mid_R	–4.48	–0.52	8.85E–06
Cingulate_Ant_L	Temporal_Pole_Mid_R	–4.34	–0.5	1.63E–05
Frontal_Inf_Orb_2_L	Postcentral_L	4.15	0.48	3.85E–05
Precuneus_R	Temporal_Inf_L	–4.12	–0.47	4.37E–05
Postcentral_L	Postcentral_R	–4	–0.46	7.01E–05
Pallidum_R	Temporal_Mid_L	3.99	0.46	7.50E–05

To address the large age span, we re-analyzed our main results of neural biomarkers with an age-stratified approach by partitioning individuals into three age bands (i.e., children, age 6–12; adolescents, age 12–18; adults, age 18–30) and found no apparent difference across the three age bands according to their effect sizes (i.e., Cohen’s *D*), except for the negative FC between the trans-hemisphere PCGs, which showed a steadily enlarged difference between the ASD-severe and controls along with the increase of age (see Supplementary Tables 9A,B, for details). Therefore, most neural biomarkers identified in the present study are universally valid for all age bands. For the sex differences, while females were relatively rare in both ASD groups (seven out of 91 in the severe ASD group and 16 out of 169 in the mild-ASD group) and therefore unlikely to provide any meaningful statistical inference, we conducted a gender-stratified analysis nevertheless and observed no clear difference between both genders (see Supplementary Tables 10A, B). Further, we re-assessed the main results with the cutoff of mean framewise displacement at 0.2 mm, and all significance remained, without a clear difference from the original results (see Supplementary Tables 11A, B).

Alternatively, we also partitioned the high-functioning autistic patients into two subgroups with thresholds of ADOS total score at 11 and 12, which, as shown above, is in line with recommended clinical cutoffs as well as balancing the sensitivity and specificity according to our FC-based nonparametric partition. With both ADOS thresholds, we failed to observe the same significant difference in strength between the severe and mild groups for the ASD-severe specific FCs (*t* = 1.78, Cohen’s *D* = 0.24, *P* = 0.077 for cutoff at 11; *t* = 0.78, Cohen’s *D* = 0.11, *P* = 0.434 for cutoff at 12; Supplementary Table 12) with much reduced effect sizes if compared with our FC-based partition (Cohen’s *D* = 0.63, Supplementary Table 6). Univariately, only two FCs between the left amygdala and left Heschl’s area and between the right pallidum and left MTG remained nominally significant (Supplementary Table 13) down from seven FCs with the FC-based partition (Supplementary Table 6). However, ASD-mild specific FCs were observed with significant stronger strength in the mild group than in the severe group with ADOS cutoff at 12 (*t* = 2.26, Cohen’s *D* = 0.31, *P* = 0.025, Supplementary Table 12), similar to the FC-based partition (Cohen’s *D* = 0.32, Supplementary Table 6) but not at 11 (*t* = 1.89, Cohen’s *D* = 0.26, *P* = 0.061, Supplementary Table 12). Univariately, only the FC between the left SFG and left MFG remains nominally significant with ADOS cutoff at 12 (Supplementary Table 14).

### Functional Connectivity-Based Prediction of ADOS Scores in High-Functioning ASD Patients

The top 20 FCs that were mostly different between the ASD-mild and ASD-severe subgroups (*P* < 0.05 after FDR correction for the 100 top-selected FCs) are shown in Table 2C. Based on these 20 FCs, our trained model of SVM with default parameters through a 10-fold strategy resulted in an inner sample correlation with a moderate effect size between the predicted and observed scores (*r* = 0.30, *t* = 4.99, *P*_one–tailed_ < 0.001, *n* = 260).

**TABLE 2C:** Biomarker of the severity of high-functioning ASD.

Functional connectivity		*t*	Cohen’s *D*	*P*_two–tailed_
Region 1	Region 2			
Frontal_Sup_Medial_L	Postcentral_R	5.42	0.74	1.44E−07
Supp_Motor_Area_L	Temporal_Mid_L	3.85	0.53	1.51E−04
Frontal_Sup_Medial_R	Postcentral_R	3.58	0.49	4.15E−04
Cingulate_Ant_R	Postcentral_R	3.53	0.48	4.95E−04
Frontal_Inf_Orb_2_L	Paracentral_Lobule_L	3.51	0.48	5.33E−04
Frontal_Mid_2_L	SupraMarginal_R	3.33	0.46	1.01E−03
Precentral_L	Precuneus_R	–3.26	–0.45	1.29E−03
Frontal_Sup_Medial_L	Temporal_Sup_L	3.24	0.44	1.37E−03
Cingulate_Post_R	Pallidum_R	3.09	0.42	2.23E−03
Insula_L	Cingulate_Post_R	3.07	0.42	2.43E−03
Frontal_Inf_Tri_L	Pallidum_R	3.01	0.41	2.89E−03
Cingulate_Ant_R	Amygdala_R	2.99	0.41	3.10E−03
Occipital_Inf_L	Temporal_Pole_Sup_L	2.96	0.4	3.42E−03
Frontal_Mid_2_R	Heschl_L	2.93	0.4	3.68E−03
Insula_R	Fusiform_R	–2.88	–0.39	4.30E−03
Precentral_R	Frontal_Sup_Medial_L	2.8	0.38	5.51E−03
Angular_R	Pallidum_R	2.8	0.38	5.62E−03
Postcentral_R	Temporal_Mid_L	2.66	0.36	8.38E−03
Frontal_Inf_Oper_R	OFCpost_L	–2.6	–0.36	9.84E−03
Frontal_Inf_Tri_L	OFCant_L	–2.6	–0.36	9.87E−03

*ASD, autism spectrum disorder.*

Next, by applying the trained SVM in the ASD validation group (i.e., with ADOS total scores but incomplete subscale information; see section “Materials and Methods” for more details; Supplementary Figure 3), we also observe a significant correlation with median strength between the predicted and observed ADOS total scores (*r* = 0.35, *t* = 1.95, *P*_one–tailed_ = 0.031, *n* = 29), thus confirming the reliability of the trained SVM model as well as its underlying FCs (Table 2C).

## Discussion

The current study aimed to identify objective neural biomarkers based on FC characteristics to stratify a highly heterogeneous neurodevelopmental condition (ASD) into more homogeneous subgroups. Through a newly developed statistical approach combining CCA and hierarchical clustering to identify candidate neural features that were further trained and independently validated with a machine learning model, our results demonstrated that rsfMRI FC-based stratification of high-functioning ASD patients was effective in differentiating severe from mild ASD patients, showing excellent consistency with traditional behavior-based diagnostic segregations at varied cutoffs of the ADOS scores. Interestingly, the FC strengths not only showed a graded change between the two ASD subgroups and healthy controls, but distinctive patterns of FCs also emerged between the severe and mild ASD subgroups. Our prediction model also showed a moderate effect in predicting individual’s ADOS scores based on the strength of FCs, providing evidence of the neural basis for a widely used clinical scale. Additionally, we found a potential compensatory role of frontal cortical areas in the mild subgroup, which might have clinical implications.

The successful partition of severe versus mild ASD patients solely based on FCs was highly consistent with the traditional diagnostic criteria using ADOS scores, showing highly consistent sensitivity and specificity no matter which ADOS cutoff was set as the reference, which therefore suggests that our FC-based nonparametric partition may serve as a better representation of the true partition (i.e., the real segregation of severe vs. mild ASD patients) than using varied cutoffs of the ADOS score.

This set of results provided scientific evidence for the potential neural basis of clinical stratification of severe and mild ASD patients, as well as suggesting candidate neural biomarkers that can be used to diagnose subgroups of ASD patients effectively. We further established an SVM model based on FCs that are mostly attributed to the “mild” versus “severe” partition. This model showed a good fitness of inner-sample prediction of ADOS total score (*r* = 0.30) under a 10-fold strategy and has also been successfully validated in an independent sample (*r* = 0.35). These effect sizes suggested that the predictors are only moderate, although those effect sizes are still considered relatively large in fMRI studies (Clements et al., 2018), thus confirming the potential role of neural biomarkers in etiological pathways for ASD severity.

The current investigation also identified two sets of univariate stratification neural biomarkers that were most significantly different between high-functioning ASD patients and controls yet specific to each of the severe and mild subgroups of ASD patients. In particular, seven FCs involving the temporal areas, amygdala, ACC, PCG, and left IFG were found to be most prominent in the ASD-severe subgroup, became weaker in the ASD-mild subgroup, and then were the weakest in healthy controls (Figures 3C,D, Table 2B, and Supplementary Table 8), thus providing additional evidence to support the FC-based severe versus mild segregation of high-functioning ASD patients. We further showed that such a graded change in strength could not be observed in traditional partitions based on thresholding the ADOS total score (Supplementary Table 12), thus indicating the advantages of our FC-based partition in the clinical diagnosis of severe versus mild autism. Notably, FC between the left amygdala and left Heschl’s area did not follow this graded pattern (Figure 3D and Supplementary Table 8), hence suggesting that the amygdala might only be involved at a later and more severe developmental stage of ASD, which is additionally supported by partitions based on ADOS total score (Supplementary Table 13). It is notable that all these areas, i.e., the temporal areas (particularly the superior temporal sulcus), amygdala, ACC, and left IFG, have been proposed as part of the “social brain” (Frith, 2007; Adolphs, 2009) and hence are in line with suggested ROIs for ASD (Cheng et al., 2015, 2017; Holiga et al., 2019).

On the other hand, three FCs between the prefrontal areas, PCG, and thalamus were found to exhibit a rather different pattern, where these FCs were, in fact, the strongest among the ASD-mild subgroup, weaker in the ASD-severe subgroup, and then the weakest in healthy controls, suggesting that these may be candidate neural biomarkers for mild ASD specifically (Figure 3A, Table 2A, and Supplementary Table 7), in particular the FC between the left SFG and the left MFG (Figure 3A and Supplementary Table 7). While almost identical results could be achieved with partition based on the ADOS total score only at a certain threshold (i.e., 12, also see Supplementary Tables 12, 14), the FC-based partition is free from the uncertainty in choosing the optimal threshold for the ADOS total score, which could vary across different age bands (Lord et al., 2000) but could converge when investigating with neural biomarkers. Nevertheless, this result suggests a compensatory role of frontal areas such that a strengthened dorsolateral prefrontal cortex (DLPFC) region might help to reduce the symptoms in ASD patients, and such a claim is further strengthened by the previous findings that repetitive transcranial magnetic stimulation (rTMS) at DLPFC could indeed improve relevant behaviors in ASD patients (Sokhadze et al., 2009, 2010, 2012; Casanova et al., 2012; Oberman et al., 2015). Therefore, we have established the neural basis for the centrality role of DLPFC as a treatment target, especially in those with severe symptoms. This promoted both noninvasive cortical stimulation to strengthen the connectivity of this area and behavioral treatment to improve cognitive function, including top-down cognitive control by the DLPFC over behavior (Elliott, 2003).

It is a limitation that although AAL series parcellation scheme has good performance of analysis in FC in the past (Tao et al., 2013; Cheng et al., 2019), this structure-based parcellation may not fully incorporate the functional architecture of the human brain. We also acknowledge that the results in this study may only apply to individuals with ASD and without comorbid intellectual disability, as only high-functioning ASD patients have been investigated.

## Conclusion

In conclusion, we have provided multiple pieces of evidence, with validation, to show that FCs of rsfMRI underlie the neural basis of ASD severity. Specifically, we used FCs to partition high-functioning autistic individuals into severe versus mild subgroups, which exhibited advantages over the traditional clinical partition based on the ADOS total score. In addition, we showed that FCs could predict the ADOS total score in high-functioning autistic individuals, as well as distinguish ASD patients from controls. In addition, the strengthened FCs in the prefrontal areas that are specific to the mild ASD may provide a compensatory mechanism for the severity of ASD and thus indicate promising targets for future clinical treatments.

## Data Availability Statement

The original contributions presented in the study are included in the article/Supplementary Material, further inquiries can be directed to the corresponding authors.

## Ethics Statement

The studies involving human participants were reviewed and approved by the respective site Institutional Review Board (IRB). Written informed consent to participate in this study was provided by the participants’ legal guardian/next of kin.

## Author Contributions

DC and TJ contributed equally to the data analysis and writing of the manuscript. YZ, EL, and BS contributed to the writing of the manuscript. DC, WC, ZL, MC, C-YL, and WG contributed to the data collection and data preprocessing. DC, TJ, and JF contributed to the study design. All authors contributed to the article and approved the submitted version.

## Conflict of Interest

The authors declare that the research was conducted in the absence of any commercial or financial relationships that could be construed as a potential conflict of interest.

## Footnotes

1
http://fcon_1000.projects.nitrc.org/indi/abide/

2 https://github.com/weikanggong/Resting-state-fMRI-preprocessing

3 https://fsl.fmrib.ox.ac.uk/fsl/fslwiki/FLIRT

4 https://fsl.fmrib.ox.ac.uk/fsl/fslwiki/FNIRT

5 https://fsl.fmrib.ox.ac.uk/fsl/fslwiki/FAST
