# Supplementary material for: Neural Biomarkers Distinguish Severe From Mild Autism Spectrum Disorder Among High-Functioning Individuals
Source: Front Hum Neurosci. 2021 May 6;15:657857. doi: 10.3389/fnhum.2021.657857 (PMC8134539; doi:10.3389/fnhum.2021.657857)
Supplement: Supplementary file 1 [file Data_Sheet_1.pdf]

Supplementary Information

Supplementary Tables

Supplementary Table 1. The automated anatomical labelling atlas AAL2.

Supplementary Table 1 is taken from a previously published paper by Cheng et al. **(Cheng et al., 2019)**

**NO.**	**ANATOMICAL DESCRIPTION**	**LABEL aal2.nii.gz**	**POSSIBLE ABBREVIATION**
1,2	Precentral gyrus	Precentral	PreCG
3, 4	Superior frontal gyrus, dorsolateral	Frontal_Sup	SFG
5, 6	Middle frontal gyrus	Frontal_Mid	MFG
7, 8	Inferior frontal gyrus, opercular part	Frontal_Inf_Oper	IFGoperc
9, 10	Inferior frontal gyrus, triangular part	Frontal_Inf_Tri	IFGtriang
11, 12	IFG pars orbitalis,	Frontal_Inf_Orb	IFGorb
13, 14	Rolandic operculum	Rolandic_Oper	ROL
15, 16	Supplementary motor area	Supp_Motor_Area	SMA
17, 18	Olfactory cortex	Olfactory	OLF
19, 20	Superior frontal gyrus, medial	Frontal_Sup_Med	SFGmedial
21, 22	Superior frontal gyrus, medial orbital	Frontal_Med_Orb	PFCventmed
23, 24	Gyrus rectus	Rectus	REC
25, 26	Medial orbital gyrus	OFCmed	OFCmed
27, 28	Anterior orbital gyrus	OFCant	OFCant
29, 30	Posterior orbital gyrus	OFCpost	OFCpost
31, 32	Lateral orbital gyrus	OFClat	OFClat
33, 34	Insula	Insula	INS
35, 36	Anterior cingulate & paracingulate gyri	Cingulate_Ant	ACC
37, 38	Middle cingulate & paracingulate gyri	Cingulate_Mid	MCC
39, 40	Posterior cingulate gyrus	Cingulate_Post	PCC
41, 42	Hippocampus	Hippocampus	HIP
43, 44	Parahippocampal gyrus	ParaHippocampal	PHG
45, 46	Amygdala	Amygdala	AMYG
47, 48	Calcarine fissure and surrounding cortex	Calcarine	CAL
49, 50	Cuneus	Cuneus	CUN
51, 52	Lingual gyrus	Lingual	LING
53, 54	Superior occipital gyrus	Occipital_Sup	SOG
55, 56	Middle occipital gyrus	Occipital_Mid	MOG
57, 58	Inferior occipital gyrus	Occipital_Inf	IOG
59, 60	Fusiform gyrus	Fusiform	FFG
61, 62	Postcentral gyrus	Postcentral	PoCG
63, 64	Superior parietal gyrus	Parietal_Sup	SPG
65, 66	Inferior parietal gyrus, excluding supramarginal and angular gyri	Parietal_Inf	IPG
67, 68	SupraMarginal gyrus	SupraMarginal	SMG
69, 70	Angular gyrus	Angular	ANG
71, 72	Precuneus	Precuneus	PCUN
73, 74	Paracentral lobule	Paracentral_Lobule	PCL
75, 76	Caudate nucleus	Caudate	CAU
77, 78	Lenticular nucleus, Putamen	Putamen	PUT
79, 80	Lenticular nucleus, Pallidum	Pallidum	PAL
81, 82	Thalamus	Thalamus	THA
83, 84	Heschl's gyrus	Heschl	HES

85, 86	Superior temporal gyrus	Temporal_Sup	STG
87, 88	Temporal pole: superior temporal gyrus	Temporal_Pole_Sup	TPOsup
89, 90	Middle temporal gyrus	Temporal_Mid	MTG
91, 92	Temporal pole: middle temporal gyrus	Temporal_Pole_Mid	TPOmid
93, 94	Inferior temporal gyrus	Temporal_Inf	ITG

Supplementary Table 2. Clinical symptoms

Supplementary Table 2 comes from: http://fcon_1000.projects.nitrc.org/indi/abide/

ABIDE II LABEL	ABIDE II DESCRIPTION	ABIDE I LABEL	ABIDE I DESCRIPTION
ADOS_G_TOTAL	ADOS Generic or ADOS-2 Module 4 Total Score	ADOS_TOTAL	Classic Total ADOS Score (Communication subscore + Social Interaction subscore)
ADOS_G_COMM	ADOS-G or ADOS-2 Module 4 Communication Total	ADOS_COMM	Communication Total Subscore of the Classic ADOS
ADOS_G_SOCIAL	ADOS-G or ADOS-2 Module 4 Reciprocal Social Interaction Total	ADOS_SOCIAL	Social Total Subscore of the Classic ADOS
ADOS_G_STEREO_ BEHAV	ADOS-G or ADOS-2 Module 4 Stereotyped Behaviors and Restricted Interest Total	ADOS_STERO_ BEHAV	Sterotyped Behaviors and Restricted Interests Total Subscore of the Classic ADOS

Supplementary Table 3. The analysis of different thresholds of P-values

Threshold	number of features	feature to sample radio	CCA performance
p<0.05	724	2.7846	R1=1.00; R2=1.00; R3=1.00
p<0.005 (Ours)	100	0.3846	R1=0.79; R2=0.78; R3=0.70
p<0.001	19	0.0731	R1=0.62; R2=0.54; R3=0.33

Supplementary Table 4. Mean of silhouette values by number of clusters

The optimal number of clusters was detected as 2, i.e. the highest mean silhouette score (Metric: Squared Euclidean distance).

Number of clusters	Mean of silhouette score
2	0.3088
3	0.1234
4	0.1785
5	0.2273
6	0.1416
7	0.1739
8	0.2054
9	0.2240
10	0.2244

Supplementary Table 5. Accuracy, Sensitivity and Specificity

Cutoff	Accuracy	Ground truth: fMRI Classification		Ground truth: Varied ADOS Cut-offs	
		**Sensitivity**	**Specificity**	**Sensitivity**	**Specificity**
8	43.46%	0.9670	0.1479	0.3793	0.8929
9	53.46%	0.9341	0.3195	0.4250	0.9000
10	60.77%	0.8352	0.4852	0.4663	0.8454
11	66.92%	0.7582	0.6213	0.5188	0.8268
12	69.23%	0.6264	0.7278	0.5534	0.7834
13	71.92%	0.5385	0.8166	0.6125	0.7667
14	75.00%	0.4505	0.9112	0.7321	0.7549
15	75.00%	0.3736	0.9527	0.8095	0.7385
16	71.54%	0.2527	0.9645	0.7931	0.7056
17	70.77%	0.1868	0.9882	0.8947	0.6929
18	67.31%	0.0769	0.9941	0.8750	0.6667
19	66.15%	0.0330	1.0000	1.0000	0.6576
20	65.77%	0.0220	1.0000	1.0000	0.6550
21	65.38%	0.0110	1.0000	1.0000	0.6525

Supplementary Table 6. Tendency analysis of systematic pattern

	Severe vs Mild		Severe vs Control		Mild vs Control	
	**T**	$P_{two\text{-}tailed}$	**T**	$P_{two\text{-}tailed}$	**T**	$P_{two\text{-}tailed}$
**Severe systematic pattern**	4.63	<0.001	9.87	<0.001	4.50	<0.001
**Mild systematic pattern**	-2.35	0.020	2.94	0.003	7.25	<0.001

Supplementary Table 7. Tendency analysis of mild biomarker

Functional Connectivity		Severe vs Mild		Severe vs Control		Mild vs Control	
Region 1	Region 2	T	$P_{one-tailed}$	T	$P_{one-tailed}$	T	$P_{one-tailed}$
Postcentral_L	Thalamus_R	-0.60	0.273	2.18	0.015	4.38	<0.001
Frontal_Sup_2_L	Frontal_Mid_2_L	-1.90	0.029	1.68	0.046	4.36	<0.001
Hippocampus_R	Precuneus_L	1.82	0.035	-1.47	0.071	-4.25	<0.001

Supplementary Table 8. Tendency analysis of severe biomarker

Functional Connectivity		Severe vs Mild		Severe vs Control		Mild vs Control	
Region 1	Region 2	T	$P_{one-tailed}$	T	$P_{one-tailed}$	T	$P_{one-tailed}$
Amygdala_L	Heschl_L	-2.62	0.005	-4.54	<0.001	-0.64	0.262
Cingulate_Ant_L	Temporal_Mid_R	-1.73	0.042	-4.48	<0.001	-2.25	0.012
Cingulate_Ant_L	Temporal_Pole_Mid_R	-0.68	0.248	-4.34	<0.001	-3.02	0.001
Frontal_Inf_Orb_2_L	Postcentral_L	2.43	0.008	4.15	<0.001	1.78	0.038
Precuneus_R	Temporal_Inf_L	-2.36	0.010	-4.12	<0.001	-0.68	0.248
Postcentral_L	Postcentral_R	-1.27	0.104	-4.00	<0.001	-2.95	0.002
Pallidum_R	Temporal_Mid_L	2.84	0.002	3.99	<0.001	1.54	0.062

Supplementary Table 9. A.
ASD-severe biomarkers with an age-stratified approach (ASD-severe vs. Controls)

Functional Connectivity		Full sample (original result) (n=91 vs. n=574)	Children (age: 6-12) (n=35 vs. n=323)	Adolescents (age: 12-18) (n=31 vs. n=159)	Adults (age: 18-30) (n=25 vs. n=92)
Region 1	Region 2	Cohen's D	Cohen's D	Cohen's D	Cohen's D
Amygdala_L	Heschl_L	-0.52	-0.57	-0.56	-0.46
Cingulate_Ant_L	Temporal_Mid_R	-0.52	-0.53	-0.47	-0.45
Cingulate_Ant_L	Temporal_Pole_Mid_R	-0.50	-0.44	-0.58	-0.75
Frontal_Inf_Orb_2_L	Postcentral_L	0.48	0.68	0.14	0.46
Precuneus_R	Temporal_Inf_L	-0.47	-0.35	-0.38	-0.67
Postcentral_L	Postcentral_R	-0.46	-0.02	-0.60	-1.04
Pallidum_R	Temporal_Mid_L	0.46	0.33	0.74	0.45

Supplementary Table 9. B.
ASD-mild biomarkers with an age-stratified approach (ASD-mild vs. Controls)

Functional Connectivity		Full sample (original result) (n=169 vs. n=574)	Children (age: 6-12) (n=65 vs. n=323)	Adolescents (age: 12-18) (n=55 vs. n=159)	Adults (age: 18-30) (n=49 vs. n=92)
Region 1	Region 2	Cohen's D	Cohen's D	Cohen's D	Cohen's D
Postcentral_L	Thalamus_R	0.39	0.38	0.29	0.53
Frontal_Sup_2_L	Frontal_Mid_2_L	0.39	0.38	0.41	0.71
Hippocampus_R	Precuneus_L	-0.38	-0.47	-0.32	-0.15

Supplementary Table 10. A. The analysis for the sex differences of ASD-severe biomarkers (ASD-severe vs. Controls)

Functional Connectivity		Male & Female (original result) (n=91 vs. n=574)			Female only (n=7 vs. n=169)			Male only (n=84 vs. n=405)		
**Region 1**	**Region 2**	**T**	**Cohen's D**	**P**	**T**	**Cohen's D**	**P**	**T**	**Cohen's D**	**P**
Amygdala_L	Heschl_L	-4.54	-0.52	6.79E-06	-1.35	-0.56	1.79E-01	-4.15	-0.51	4.02E-05
Cingulate_Ant_L	Temporal_Mid_R	-4.48	-0.52	8.85E-06	-1.65	-0.68	1.01E-01	-3.88	-0.48	1.20E-04
Cingulate_Ant_L	Temporal_Pole_Mid_R	-4.34	-0.50	1.63E-05	-1.92	-0.80	5.65E-02	-3.83	-0.47	1.45E-04
Frontal_Inf_Orb_2_L	Postcentral_L	4.15	0.48	3.85E-05	0.74	0.31	4.62E-01	3.83	0.47	1.48E-04
Precuneus_R	Temporal_Inf_L	-4.12	-0.47	4.37E-05	-1.09	-0.45	2.77E-01	-4.13	-0.51	4.27E-05
Postcentral_L	Postcentral_R	-4.00	-0.46	7.01E-05	-0.39	-0.16	6.98E-01	-3.96	-0.49	8.54E-05
Pallidum_R	Temporal_Mid_L	3.99	0.46	7.50E-05	1.52	0.63	1.30E-01	3.72	0.46	2.22E-04

Supplementary Table 10. B. The analysis for the sex differences of ASD-mild biomarkers (ASD-mild vs. Controls)

Functional Connectivity		Male & Female (original result) (n=169 vs. n=574)			Female only (n=16 vs. n=169)			Male only (n=153 vs. n=405)		
**Region 1**	**Region 2**	**T**	**Cohen's D**	**P**	**T**	**Cohen's D**	**P**	**T**	**Cohen's D**	**P**
Postcentral_L	Thalamus_R	4.38	0.39	1.39E-05	1.50	0.42	1.35E-01	3.90	0.38	1.09E-04
Frontal_Sup_2_L	Frontal_Mid_2_L	4.36	0.39	1.48E-05	0.30	0.08	7.66E-01	4.58	0.44	5.68E-06
Hippocampus_R	Precuneus_L	-4.25	-0.38	2.45E-05	-1.00	-0.28	3.20E-01	-4.36	-0.42	1.56E-05

Supplementary Table 11. A. The analysis for the different mean-FD's cut-offs of ASD-severe biomarkers (ASD-severe vs. Controls)

Functional Connectivity		Cutoff=0.5mm (original result) (n=91 vs. n=574)			Cutoff=0.2mm (n=73 vs. n=486)		
Region 1	Region 2	T	Cohen's D	P	T	Cohen's D	P
Amygdala_L	Heschl_L	-4.54	-0.52	6.79E-06	-4.14	-0.53	4.01E-05
Cingulate_Ant_L	Temporal_Mid_R	-4.48	-0.52	8.85E-06	-3.50	-0.45	5.11E-04
Cingulate_Ant_L	Temporal_Pole_Mid_R	-4.34	-0.50	1.63E-05	-3.48	-0.45	5.42E-04
Frontal_Inf_Orb_2_L	Postcentral_L	4.15	0.48	3.85E-05	4.18	0.54	3.34E-05
Precuneus_R	Temporal_Inf_L	-4.12	-0.47	4.37E-05	-4.42	-0.57	1.18E-05
Postcentral_L	Postcentral_R	-4.00	-0.46	7.01E-05	-4.20	-0.54	3.09E-05
Pallidum_R	Temporal_Mid_L	3.99	0.46	7.50E-05	3.41	0.44	7.05E-04

Supplementary Table 11. B. The analysis for the different mean-FD's cut-offs of ASD-mild biomarkers (ASD-mild vs. Controls)

Functional Connectivity		Cutoff=0.5mm (original result) (n=169 vs. n=574)			Cutoff=0.2mm (n=129 vs. n=486)		
Region 1	Region 2	T	Cohen's D	P	T	Cohen's D	P
Postcentral_L	Thalamus_R	4.38	0.39	1.39E-05	3.87	0.39	1.19E-04
Frontal_Sup_2_L	Frontal_Mid_2_L	4.36	0.39	1.48E-05	4.92	0.50	1.10E-06
Hippocampus_R	Precuneus_L	-4.25	-0.38	2.45E-05	-4.31	-0.44	1.95E-05

Supplementary Table 12. Systematic pattern between severe vs mild group with varied cut-off on the ADOS total score

	ADOS Cutoff = 11		ADOS Cutoff = 12	
	T	$P_{two-tailed}$	T	$P_{two-tailed}$
**Severe systematic pattern**	1.78	0.077	0.78	0.434
**Mild systematic pattern**	-1.89	0.061	-2.26	0.025

Supplementary Table 13. Severe biomarker between severe vs mild group with varied cut-off on the ADOS total score

Functional Connectivity		ADOS Cutoff = 11		ADOS Cutoff = 12	
**Region 1**	**Region 2**	**T**	**$P_{one\text{-}tailed}$**	**T**	**$P_{one\text{-}tailed}$**
Amygdala_L	Heschl_L	-2.56	0.006	-1.70	0.045
Cingulate_Ant_L	Temporal_Mid_R	-0.37	0.357	-0.47	0.319
Cingulate_Ant_L	Temporal_Pole_Mid_R	-0.58	0.280	-0.62	0.269
Frontal_Inf_Orb_2_L	Postcentral_L	0.52	0.301	-0.84	0.900
Precuneus_R	Temporal_Inf_L	-0.82	0.205	-0.10	0.459
Postcentral_L	Postcentral_R	0.92	0.911	1.01	0.922
Pallidum_R	Temporal_Mid_L	2.04	0.021	1.68	0.047

Supplementary Table 14. Mild biomarker between severe vs mild group with varied cut-off on the ADOS total score

Functional Connectivity		ADOS Cutoff = 11		ADOS Cutoff = 12	
**Region 1**	**Region 2**	**T**	**$P_{one\text{-}tailed}$**	**T**	**$P_{one\text{-}tailed}$**
Postcentral_L	Thalamus_R	-1.45	0.074	-1.18	0.120
Frontal_Sup_2_L	Frontal_Mid_2_L	-1.11	0.134	-1.77	0.039
Hippocampus_R	Precuneus_L	0.99	0.161	1.22	0.112

Supplementary Figure

Supplementary Figure 1. Heatmap of Original FCs

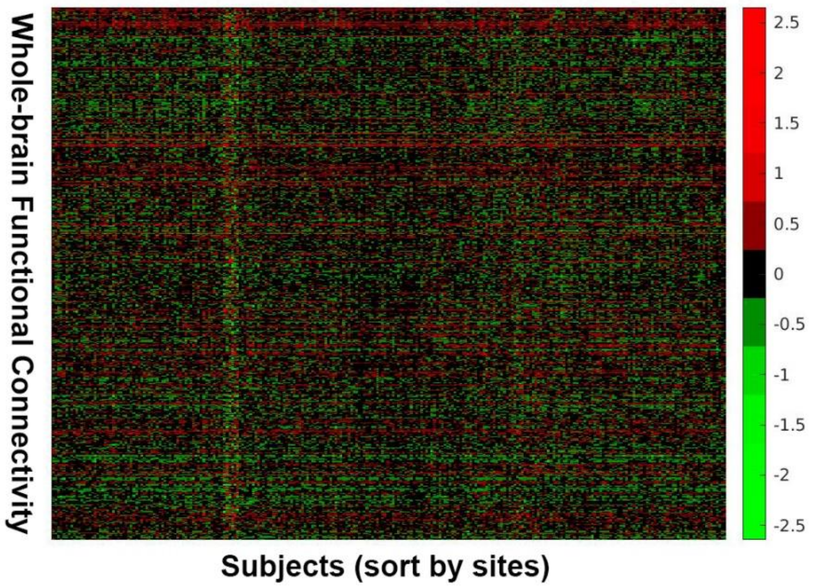

Original FCs
(The discovery ASD subjects were sorted by sites, n=260)

Supplementary Figure 2. FCs after normalization and covariate-regression

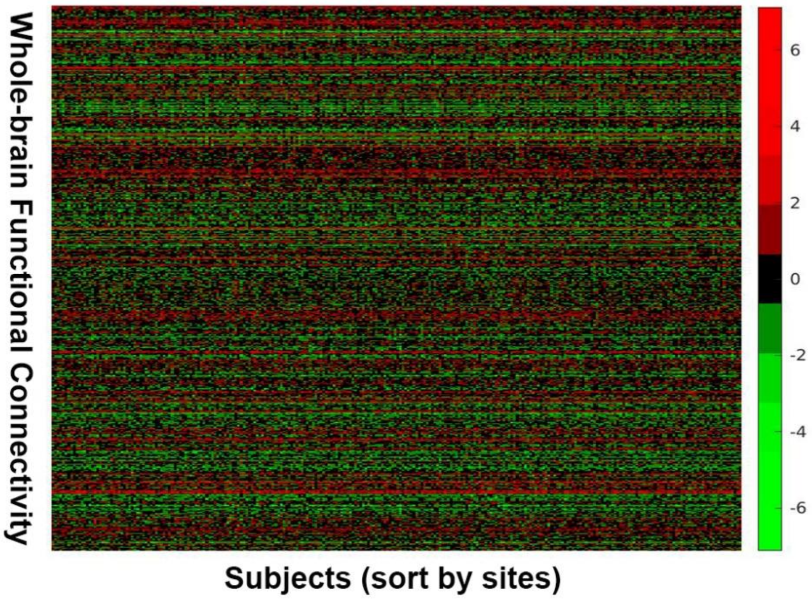

FCs after normalization and covariate-regression
(The discovery ASD subjects were sorted by sites, n=260)

Supplementary Figure 3. Flowchart of the Support Vector Machine model.

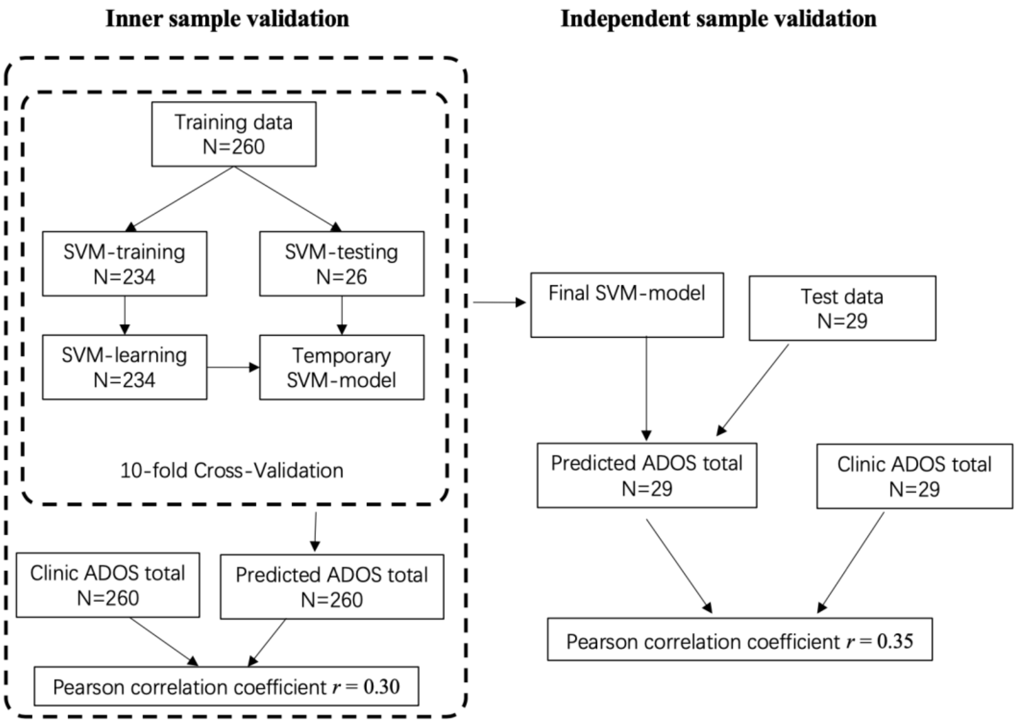

Supplementary Reference

Cheng, W., Rolls, E.T., Robbins, T.W., Gong, W.K., Liu, Z.W., Lv, W.J., Du, J.N., Wen, H.K., Ma, L., Quinlan, E.B., Garavan, H., Artiges, E., Orfanos, D.P., Smolka, M.N., Schumann, G., Kendrick, K., and Feng, J.F. (2019). Decreased brain connectivity in smoking contrasts with increased connectivity in drinking. *Elife* 8.
